# COGNITION AND HEALTH: PRELIMINARY RESULTS OF A MUSIC CREATIVITY INTERVENTION FOR ADULTS AT RISK OF MCI

**DOI:** 10.1093/geroni/igac059.2675

**Published:** 2022-12-20

**Authors:** E Lim Lydia Wu-Chung, Anthony Brandt, Melia Bonomo, Bryan Denny, Christof Karmonik, Jefferson Frazier, Karl Blench, Christopher Fagundes

**Affiliations:** Rice University, Houston, Texas, United States; Rice University, Houston, Texas, United States; Rice University, Houston, Texas, United States; Rice University, Houston, Texas, United States; Houston Methodist Research Institute, Houston, Texas, United States; Houston Methodist Hospital, Houston, Texas, United States; Rice University, Houston, Texas, United States; Rice University, Houston, Texas, United States

## Abstract

As the number of people with dementia is projected to rise to 13.8 million by 2050, there is a growing need to develop interventions that prevent or slow down disease progression in at-risk individuals. Older adults are particularly vulnerable, given that increasing age is the strongest predictor of dementia. Music interventions are promising, non-pharmaceutical treatment options for slowing down cognitive decline and enhancing psychological health. However, more music-related clinical trials are needed to evaluate treatment efficacy and to identify biobehavioral mechanisms of change. Project Chroma is a Stage 1 semi-randomized clinical trial, developed to assess the effects of a novel, music creativity curriculum on cognitive, socio-emotional, neurobiological, and immunological outcomes. In this study of 58 older adults with or without MCI, we demonstrate that Project Chroma has good feasibility and acceptability: participation, retention and satisfaction rates were comparable to other similarly designed clinical trials. Preliminary analyses revealed that participants in the music condition, relative to those in the control condition, showed marked improvements in cognitive functioning. Slight changes in socioemotional well-being were observed, which may be attributed to a minimally distressed sample. This study contributes to a growing literature substantiating music interventions as effective options for curtailing cognitive decline. Forthcoming work will examine the effects of music creativity on neural and immune outcomes.

